# Association Study of the Beta-Adrenergic Receptor Genetic Variant Gly389Arg and Fluoxetine Response in Major Depression

**DOI:** 10.31661/gmj.v9i0.1781

**Published:** 2020-03-05

**Authors:** Negar Firouzabadi, Roja Asadpour, Kamiar Zomorrodian

**Affiliations:** ^1^Department of Pharmacology & Toxicology, School of Pharmacy, Shiraz University of Medical Sciences, Shiraz, Iran; ^2^Department of Medical Parasitology and Mycology, School of Medicine, Shiraz University of Medical Sciences, Shiraz, Iran

**Keywords:** Major Depressive Disorder, Fluoxetine, Genetic Polymorphism, Pharmacogenetics

## Abstract

**Background:**

Pharmacogenetics has proven role in the treatment of different illnesses. Patients with special genotypes may achieve a better response to a specific drug. On the other hand, genetic parameters markedly contribute to the development of major depressive disorder (MDD). The significance of adrenergic system compartments in cognition and behavior, and their role in etiology of depression denote that adrenergic receptors beta gene polymorphism(s) might also have an association with drug response. Thus this study aims to evaluate the association between β1AR gene polymorphisms, G1165C, Arg389Gly and response to fluoxetine in MDD patients.

**Materials and Methods:**

Among different antidepressants, we focused on fluoxetine as it is prescribed frequently in MDD and belongs to one of the most efficient antidepressant categories with minimum side effects. MDD was diagnosed at study entry using DSM-IV criteria. One hundred and one new MDD patients were treated with fluoxetine for a period of 6 weeks. A 50% decrease in Hamilton Rating Scale for Depression (HRSD) was considered as response to treatment. Genotyping of G1165C polymorphism was performed by PCR-RFLP method.

**Results:**

Results of the study indicated no significant relationship between β1AR polymorphism and the patient’s response to fluoxetine neither at genotypic nor allelic level (P=0.568).

**Conclusion:**

Our study did not support the hypothesis of involvement of β1AR Arg389Gly polymorphism and response to fluoxetine in MDD patients.

## Introduction

Major depressive disorder (MDD) is a serious, moderately heritable health issue imposing considerable burden to the healthcare system [1]. Lifetime prevalence of MDD varies from 8% to 12% [2,3]. Genetic parameters markedly contribute to the development of major depressive disorder (MDD). Early studies have proven a relationship between impairment of the adrenergic and serotonergic system and development of depression. Since association between neurotransmitters such as norepinephrine and depression has been widely reported [4-6] some functional polymorphisms on the beta adrenergic receptor gene might be a predictor of response antidepressant treatment. Recently several pieces of evidence suggested that depression is associated with the up-regulation of b adrenergic receptors (βAR) [7-10]. Adrenergic receptors are considered as G protein-coupled receptors being the targets for many catechol amines like norepinephrine and epinephrine, being divided into two types α and β receptors. The β-adrenergic receptors consist of three subtypes β1 (the most frequent subtype in brain), β2 and β3 [7,11,12,13]. The β 1-adrenergic receptor (β1AR) located on chromosome 10q24-q26, is a crucial surface signaling protein that modulates sympathetic nervous system actions through catecholamine release. Interestingly many studies have revealed a relationship between this region and depressive disorders [7,14]. Previous genetic studies focused on finding polymorphisms in genes associated with serotonin, norepinephrine, and dopamine neurotransmission. A functional polymorphism of G1165C (Gly389Arg) which causes an amino acid variation, was identified in the β1AR gene [15].The mutant allele of this variant enhances Gs coupling and as a result the post receptor signaling molecules [15]. Pharmacogenetics assistance in predicting antidepressant response is a promising avenue toward personalizing MDD treatment. Development of SSRIs with high efficacy and few associated side effects is an important achievement in treatment of depression [16,17]. However, while many studies have revealed reasonable response rates to treatment, remission rates remain noticeably less [16]. Considering different interindividual responses to antidepressants and significant role of genetic in pathophysiology of MDD, here in this study we assumed the possible association between βARs polymorphism Arg389Gly (G1165C) and response to fluoxetine in newly diagnosed MDD individuals.

## Materials and Methods

###  Study Population

Prior to interviewing, patients were asked to sign the written consent. Local ethics committee of Shiraz University of Medical Sciences on experiments conducted on humans approved the study protocol. Patients were recruited from Hafez Hospital, Shiraz University of Medical Sciences and followed during 6 weeks by a psychiatrist. Hundred and one newly diagnosed MDD patients (male: 40, female: 61, mean age± S.D.: 34.5 ± 6.9) were included in the study according to DSM-V criteria and by a psychiatrist [18]. Subjects with no previous history of depression and also no history of antidepressant use were considered as newly diagnosed. Exclusion criteria were: personal history of bipolar disorder, anxiety disorders, schizophrenia, episodes of mania or hypomania, psychotic symptoms, substance abuse or dependence, and current antipsychotic treatment or use of mood stabilizers and family history of bipolar disorders, major depressive disorder and schizophrenia in first-degree relatives. In order to estimate the severity of depression, 21-item Hamilton Depression Rating Scale (HDRS) was used at baseline (week 0) and after 6 weeks treatment period [19]. Ultimately, the patients were categorized into two groups of responsive and non-responsive. The level of assessment was measured based on 50% decrease in psychiatric symptoms after the course of treatment.

###  Drug Administration

 20 mg of Fluoxetine (FLUOXETINE-ABIDI®) was given to patients for one week initially. Based on clinical response of patients during the 6-week treatment, the dose was increased by 20 mg/day per week to a maximum of 80 mg/day. In case of severe anxiety only anxiolytics such as chlordiazepoxide or hypnotics such as zolpidem were allowed. In case of achieving a 50% reduction in HDRS after a 6-week treatment period, a positive response to treatment was considered. Assessments were done at the time of enrolment and week 6 of treatment. Before the interview, 5ml of blood samples were taken for DNA genotyping.

###  DNA Extraction and Genotyping

Applying a salting-out method, DNAs were extracted from whole blood leukocytes [20]. G1165C was amplified by PCR method and using the primers mentioned in Table-1. According to Moriyama *et al* previous study, G1165C was genotyped [21]. All samples were genotyped twice. The thermal cycler was an Eppendorf gradient Master cycler (Hamburg, Germany) PCR machine. PCR products (7μl) were digested by use of Mva I (TaKaRaBio). In order to separate digested fragments electrophoresis on 3 % agarose (Invitrogen® Ultra-Pure) gel was run. All fragments were stained with ethidium bromide to visualize in a UV transilluminator.

###  Statistics

 SPSS® 21.0 for windows® (SPSS Inc., Chicago, Illinois) was used for data analyses. Hardy–Weinberg equilibrium (HWE) was estimated by chi-square (χ2) test. Continuous variables are shown as mean ± S.D. Frequencies of genotype are shown in percentage (%). For testing normal distribution of continuous variables, Kolmogorov–Smirnov test was used. Pearson’s chi-square or Fisher’s exact tests were used to calculate associations between categorical variables. For estimating association between interval data Student’s t-test was performed. χ2 test was used for univariate analysis of genotypes. P-value<0.05 was considered statistically significant. To assess the association of genotype and allele frequencies with response to treatment, ORs and the corresponding 95%CI are reported.

## Results

 Among 101 enrolled participants, 57 patients (56.4%), were responders to fluoxetine (baseline HDRS: 24.26±2.13; HDRS after week 6: 8.67±2.71) and 44 patients (43.6%), were non-responders (baseline HDRS: 24.57±2.17; HDRS after week 6: 16.90±2.2). Demographic data are revealed in Table-2. No significant difference was detected between the study groups regarding sex and age (P=0.839 and 0.921 respectively). Table-3 demonstrates allele and genotype frequencies of depressed patients receiving fluoxetine based on a 50% HDRS score reduction. In patients responding to fluoxetine (N=57) genotype frequencies were 73.7% GG, 22.8% GC and 3.5% CC. In non-responders (N=44) genotype frequencies were 68.2% GG, 27.3% GC and 4.5% CC. No significant differences in genotype frequencies were found between responders and non-responders to Fluoxetine (P=0.905; OR=0.714; 95%CI=0.095-5.159). Similarly, no significant difference was noted in allele frequencies between two groups (P=0.568; OR=0.79; 95% CI=3.7-1.66).

## Discussion

According to a WHO report, depression as a global burden, affects more than 300 million people worldwide [22]. However, during the past 4 decades, developments in the pharmacologic treatment of depression have significantly affected the management of depression [23]. Nowadays pharmacogenetics has a proven role in personalized medicine. Inter-individual differences in genetic material could lead to various drug responses. Therefore, polymorphisms are useful tools in identification of these inter-individualized differences as well as selecting the drug of choice for each patient. Results of our study suggests lack of association between G1165C variants of β1AR and response to fluoxetine at both genotypic and allelic level (P=0.905; OR=0.714; 95%CI=0.095-5.159 and P=0.568; OR=0.79; 95%CI=3.7-1.66 respectively). By means of implementing pharmacogenomics in different ethnic populations, it is possible to predict the genetic predisposition of each person to various diseases and evaluate their response to different drugs [24]. Subsequently, it could give rise to high- risk patients screening and innovating effective preventive approaches. Recently, genetics not only shed light on the multifactorial nature of depression, but also implies significant effects on management of diseases and improving the quality of life. Among the adrenergic receptors, β1 receptor genes are one of the most prominent key factors in intracellular signal transduction [25]. Several pharmacologic and physiologic studies manifested the prevalence of adrenergic system components in the brain such as β1AR subtypes and the contribution of its genes in pathophysiology and management of depression [26,27]. According to previous studies, specific polymorphisms of β1AR is associated with MDD [7-10]. Conclusively, the hypothesis that there might be a relationship between β1AR polymorphism, β adrenergic system receptors up-regulation and MDD incidence may seem possible. Although many studies have proven the significant role of adrenergic system and its polymorphisms in MDD, still its role as therapeutic determinant remains unclear. In 2003 Zill *et al*. suggested that G1165C (Arg389Gly) is an important variant of β1ARs which plays a crucial role in a better and faster response to antidepressants of different classes [16]. A study by Crowley *et al*. showed lack of association between the functional polymorphism of G1165C and response to citalopram in depressed patients [28]. Another study carried out by Firouzabadi *et al*. examining the effect of the same polymorphism in response to sertraline suggested that the patients who carried CC genotype responded five times more to sertraline in comparison with other variants and carriers of C allele responded three times more to sertraline than patients with the G allele [29]. According to our knowledge, role of G1165C polymorphism in efficacy of response to fluoxetine in a set of newly diagnosed Iranian MDD patients has not been conducted to date. Although a previous study on MDD patients treated with sertraline showed a strong association between G1165C polymorphism and response to sertraline [29], our study on another antidepressant of the same class (SSRIs), fluoxetine shows no association with better response to treatment. Considering that this study also investigated one hundred MDD patients, it seems that lack of association between the mentioned polymorphism and response to fluoxetine is not due to sample size. It is worth noting that our study examined the role of only one antidepressant (Fluoxetine) from the SSRI family unlike the study performed by Zill *et al* which examined the effect of G1165C polymorphism on response to several classes of antidepressants [16]. According to the results of our study, it can be concluded that the functional G1165C (Gly389Arg) polymorphism in the β1AR is not associated with response to fluoxetine in MDD patients (P-value=0.905) although it was strongly associated with response to sertraline [29]. It seems that one polymorphism (Arg389Gly) affects response to one SSRI (sertraline) but is not associated with response to fluoxetine. It may be postulated that interindividual variations in response to different antidepressants of one pharmacological class might be in association with different genetic mutations and variations. By means of applying pharmacogenetics in treating MDD patients much smarter medication regimens can be planned. Conclusively, these studies may define the role of G1165C polymorphism in response to antidepressant outcome. To provide stronger evidence further research should be done to diagnose novel variants in the coding or promoter regions of the β1AR gene. In conclusion, we can have a better knowledge of a pharmacogenetics basis for the response to antidepressant treatment.

## Conclusion

 Our results demonstrated no significant relationship between β1AR polymorphism and patient’s response to fluoxetine. However; further investigations with different variants of ARs might be required to establish reliable data for individualized therapy in depression.

## Acknowledgement

 The authors would like to sincerely acknowledge Dr. Ali Alavi Shoushtari for his kind contribution towards patient selection.

## Conflict of Interest

 No conflict of interests is declared.

**Table 1:** PCR Condition and Location of G1165C (Arf389Gly) Polymorphism on DNA.

**Polymorphisms**	**Primer sequence (5′-3′)**	**Location**	**Restriction enzyme **	**Allele**	**DNA fragment size (bp)**	**References**
**G1165C**	F- ACGCTGGGCATCATCATGGGC R- ACATCGTCGTCGTCGTCGTCC	chromosome (10q25.3)	MvaI at 37 ^0C^/16 hrs	G C	280/52 142/138/52	(Zill et al., 2003)

**PCR: **Polymerase Chain Reaction; **F: **Forward primer; **R: **Reverse primer.

**Table 2:** Demographic Characteristics of Patients Responsive to Fluoxetine.

**Variables**	**Responders (N=57)**	**Non-responders (N=44)**	**P-value**
Sex (male/female)	22/35	18/26	0.839
Age (years)	34.4±8.7	33.1±7.8	0.921
HAMD score 1 (before treatment)	24.26±2.13	24.57±2.17	
HAMD score 2 (after treatment)	8.67±2.71	16.90±2.2	

**Table 3:** Genotype and Allele Frequencies of Responders and Non-responders to Fluoxetine Based on 50% HDRS Reduction

**Polymorphism**	**Genotype**	**Responders (N=57)**	**Non-responders (N=44)**	**P**_C_	**OR; 95% CI**
Genotypes				0.658	0.765; 0.32-1.81
	GG	42(73.7%)	30(68.2%)		
	GC	13(22.8%)	12(27.3%)		
	CC	2(3.5%)	2(4.5%)		
Alleles				0.568	0.79; 0.37-1.66
	G	97 (85.1%)	72 (81.8%)		
	C	17 (14.9%)	16 (18.2%)		

**P**_C_ : P-value for χ2 test, **OR: **Odds Ratio, **CI: **Confidence interval
